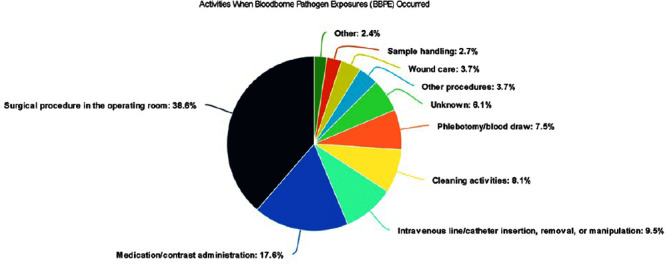# Characterization and Evaluation of a Government Health Center’s Bloodborne Pathogen Exposures (BBPE) Monitoring Program

**DOI:** 10.1017/ash.2024.277

**Published:** 2024-09-16

**Authors:** Abigail McDonald, Richard Smith, Efia James

**Affiliations:** Yale University Occupational and Environmental Medicine; VA Connecticut; VA Connecticut Healthcare

## Abstract

**Background:** A vital role of hospital employee health is the management, characterization, and targeted prevention of bloodborne pathogen exposures (BPPE) among healthcare workers. A comprehensive review of a health center’s BPPE over time was conducted to identify areas for improvement and target education and training, given changes in BBPE standard operating procedures (SOPs) over time. **Methods:** A retrospective descriptive analysis was conducted on deidentified BBPE cases reported to employee health at VA Connecticut Healthcare System from 1995-2023 (N=296) using R statistical software. **Results:** The highest number of BBPE occurred among trainee physicians (N=103, 34.8%, especially surgery and internal medicine), registered nurses (N=60, 20.3%), and non-trainee physicians (N=45, 15.2%). The most frequently implicated devices were hollow-bore (N=103, 34.8%) and suture needles (N=60, 20.3%). Most BBPE occurred during surgical procedures (N=114, 38.5%) or medication administration (N=52, 17.6%). Over half of BBPE occurred during afternoons/nights (N=172, 58.1%). Over half occurred with use of personal protective equipment (PPE) (N=181, 61.1%). The majority of BBPE implicated finger injuries (N=220, 74.3%). Blood was the most frequently reported exposure (N=127, 42.9%), a similar percentage of records did not specifically name a body fluid type (N=121) or whether PPE was used (N=110). In most cases, the source patient was identified (N=282, 95.3%) and tested (N=272, 91.9%). Forty-three sources (14.5%) had positive BBP testing, which included HIV (N=14, 4.7%), hepatitis C (N=23, 7.8%), and hepatitis B (N=6, 2.0%). Most employees presented to employee health for initial evaluation (N=231, 78%) and underwent post-exposure testing (N=266, 89.9%); most had evidence of immunity to hepatitis B (N=246, 83.1%). Eighty-three employees (28%) received HIV PEP (average=1.9 days). Most records did not indicate if this was a first-time BBPE (N=250, 84.5%). No employee records indicated seroconversion for a bloodborne pathogen. **Conclusions:** Physicians and RNs, those performing surgical procedures and administering medications, and those on second and third shifts are at highest risk and may benefit from additional interventions such as exposure assessment or education. Required recordkeeping has been variable over time. Updated national SOPs have been adapted to employee health, though additional details could be considered for quality improvement purposes, such as duration of employment, level of training, and prior BBPE prevention education. It is unclear if some information such as history of BBPE or PPE use was elicited but not documented – this information could be helpful in management of BBPEs.

**Figure:**
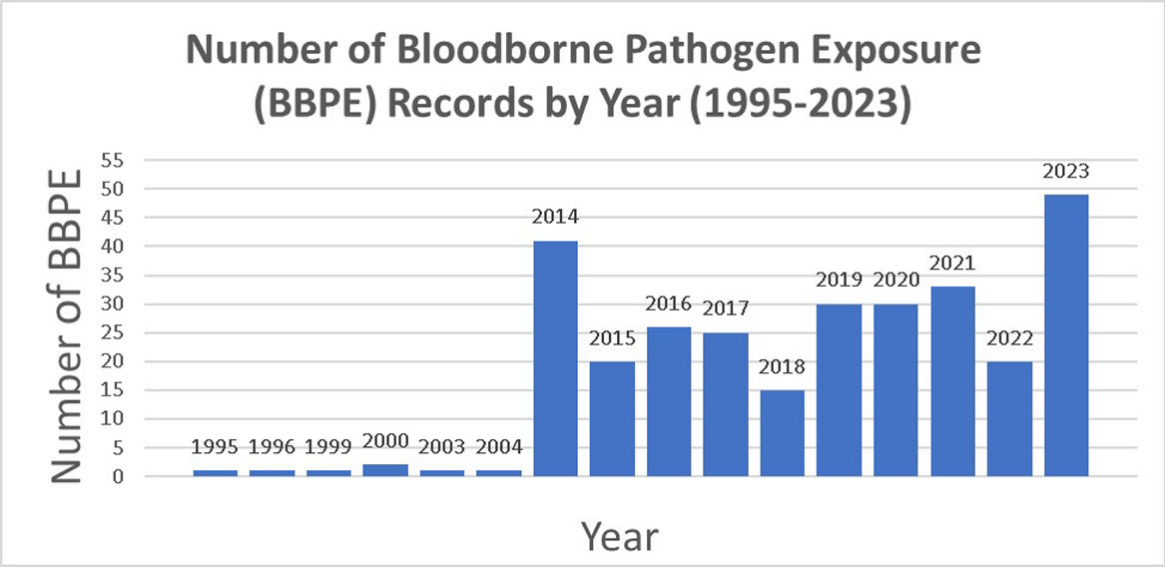

**Figure:**
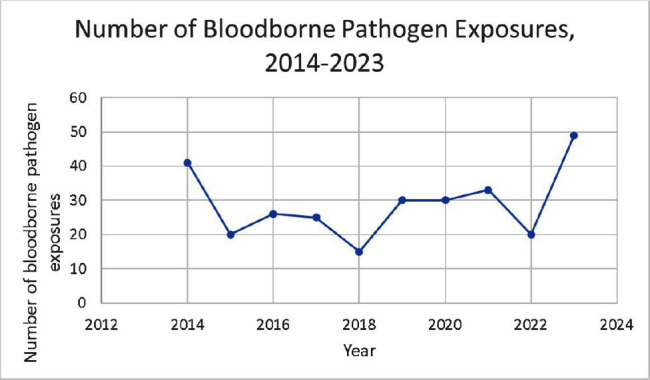

**Figure:**
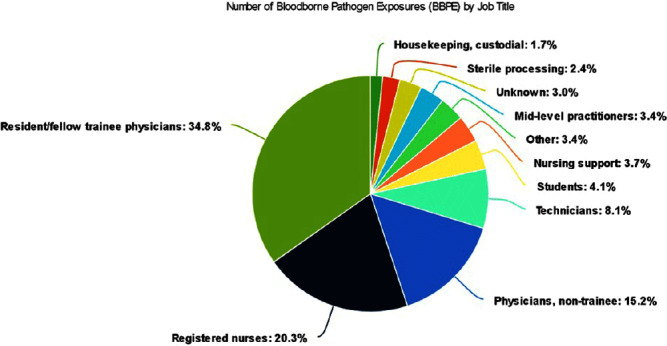

**Figure:**
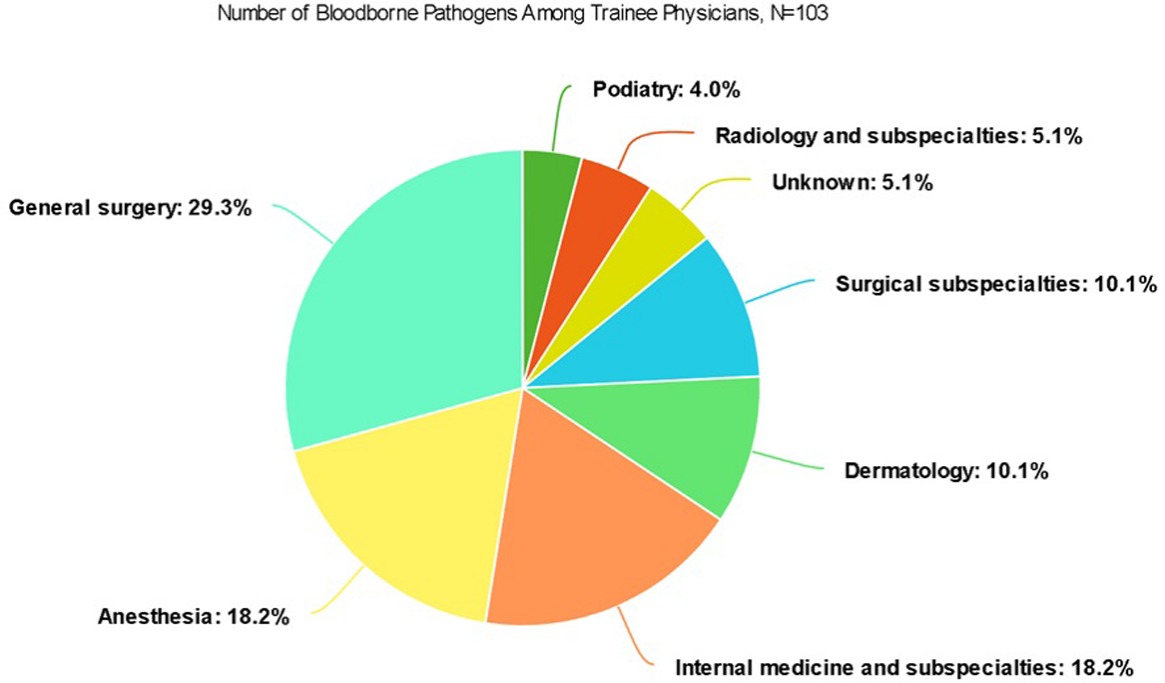

**Figure:**